# Rituximab-Induced Psoriasis in a Patient with Granulomatosis with Polyangitis Treated with Adalimumab

**DOI:** 10.1155/2019/5450863

**Published:** 2019-10-17

**Authors:** Hana S. Alahmari, Nasser Y. Alhowaish, Mohammed A. Omair

**Affiliations:** ^1^Rheumatology Unit, Department of Medicine, King Khalid University, Abha, Saudi Arabia; ^2^Rheumatology Unit, Department of Medicine, King Saud University, Riyadh, Saudi Arabia

## Abstract

Rituximab (RTX) is a chimeric B-cell-depleting monoclonal antibody against CD-20 positive cells that has been approved for the induction and maintenance of granulomatosis with polyangiitis (GPA). Reports have identified RTX to cause drug-related psoriasis. Many theories of underlying pathways have been proposed. However, further workup around the mechanism and treatment is required. Here, we present a 38-year-old woman known to have GPA that developed drug-related psoriasis that was successfully treated with treatment discontinuation and starting adalimumab along with a literature review.

## 1. Introduction

Drug-induced psoriasis is an increasingly recognized phenomenon, and different classes of therapies have been linked to drug-induced psoriasis such as antimalaria and beta-blockers [1]. Recently, biologics such as tumor necrosis factor inhibitors (TNFi), RTX, and programmed cell death protein 1 (PD-1) inhibitors have been linked to drug-related psoriasis [1]. The diagnosis is challenging for identifying the offending medication and the time lag between the onset of the rash and the drug intake. Naranjo et al. [2]. established adverse drug reaction probability scale, which would help the clinician to judge the potentiality of drug-related skin lesion such as psoriasis [1]. There are no clear specific psoriasis phenotypes provoked by the different drugs implicated in drug-related psoriasis. However, many morphological types that have been described as drug reaction included plaque psoriatic skin lesions, palmoplantar psoriasis, nail psoriasis, scalp psoriasis, pustular psoriasis, and erythrodermic psoriasis [1]. The association between B-cell depletion and the evolvement or exacerbation of psoriatic rash has been described but is not common. Such autoimmune phenomena are hypothesized to be due to the development of human antichimeric antibodies and the induction of immune-mediated skin lesions such as a psoriasiform rash [3–11] or even psoriatic arthritis (PsA) [3]. Studies are needed to identify the underlying mechanism, as well as the risk factors associated with rituximab-induced psoriatic skin lesions. Here, we present a 38-year-old woman known to have GPA that developed drug-related psoriasis along with a literature review of all cases.

## 2. Case Scenario

**Figure 1:**
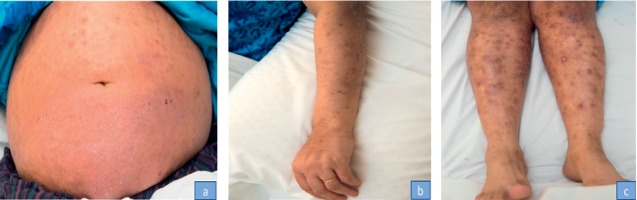
Extensive psoriasis lesions on the trunk (a), left arm (b), and legs (c).

**Figure 2:**
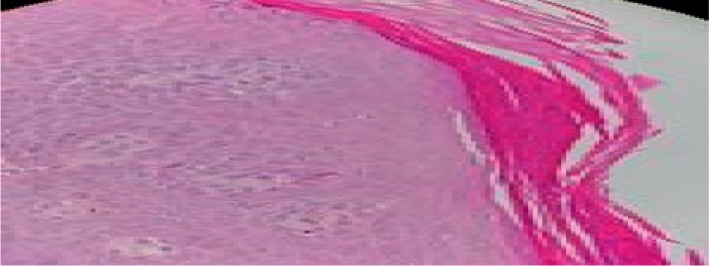
Parakeratosis, acanthosis, psoriasiform epidermal hyperplasia, and edema in capillary dermis.

**Figure 3:**
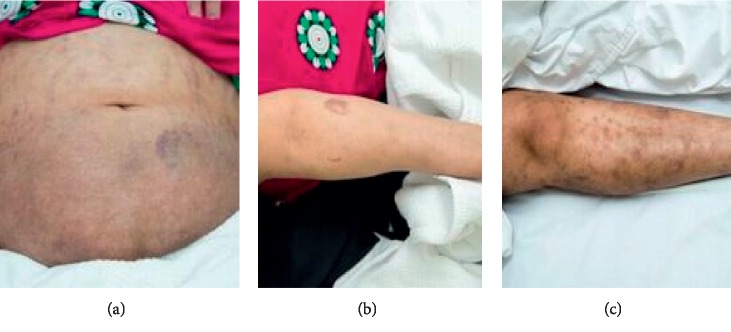
Residual hyperpigmentation without active psoriatic rash.

A 38-year-old female was diagnosed to have GPA manifested by recurrent epistaxis and one episode of pulmonary hemorrhage. Biopsy proved diffuse alveolar hemorrhage and capillaritis. She was treated with 1 gram of methylprednisolone for 3 days followed by oral prednisolone 60 mg along with 1 gram RTX infusion. No plasmapheresis was offered. She was doing well and maintaining remission on 10 mg of prednisolone and RTX courses. Three months after the third course of RTX (18 months from the first course), a scaly itchy rash erupted over the upper and lower extremities along with the abdomen. There was no joint pain or swelling. She denied the previous history of psoriatic rash, arthritis, uveitis, or chronic diarrhea. No family history of spondyloarthropathy or psoriasis was found. Examination revealed erythematous salivary scaly plaques over the abdomen (Figure 1(a)) and extensor surface of the upper (Figure 1(b)) and lower (Figure 1(c)) extremities bilaterally (sparing the hands and feet). No evidence of active synovitis or nail changes were found. The patient was evaluated by a dermatologist, and two skin biopsies were taken from the abdomen and the lateral aspect of the right leg. Hematoxylin and eosin stain revealed hyperkeratosis, focal parakeratosis, regular psoriasiform hyperplasia (Figure 2), retained granular cell layer, and superficial perivascular lymphocytes with scanty eosinophils. There was no evidence of granuloma or fungal infection. She was diagnosed to have drug-related psoriasis. She was initially treated with topical corticosteroids and psoralen and ultraviolet A (PUVA) for 3 months with interval development of new lesions and minimal response of the previously detected lesions. Since her GPA was in remission, RTX was discontinued and she was switched to subcutaneous adalimumab 40 mg every two weeks along with a topical corticosteroid. Over the next 2 months, the rash had partially improved, and no new lesion had been noticed (Figures 3(a)–3(c)).

## 3. Discussion

A total of 13 reported cases in the literature described RTX-related new onset psoriasis or psoriatic arthritis in adults. The majority had underlying rheumatoid arthritis (RA) (8 patients) [4–10], two with non-Hodgkin's lymphoma [3, 11], one with systemic lupus erythematosus [9], one with idiopathic membranous glomerulopathy [12], and one patient treated for chronic idiopathic demyelinating polyneuropathy disorder [13]. Most cases developed localized psoriasis over the hands or legs, while few developed the rash over the scalp [7, 9] or experienced pustular psoriasis in the palms and soles [6, 11]. Similar to our case, one patient [10] had a widespread psoriatic rash. The time of onset was variable, it ranged from 10 days to 2 years from the first dose. Overall, the outcome was benign, and the lesions completely or partially improved several months after discontinuation of RTX alone or in combination with topical or oral corticosteroid except in 2 cases where methotrexate was introduced in one [4] and tocilizumab in the other case [8]. Nevertheless, in our case, due to intolerable itchiness and widespread distribution, adalimumab was added. Itchiness resolved and significant improvement occurred after 4 weeks of therapy.

The mechanism by which RTX can worsen or induce psoriasis is unclear, but it may be due to several reasons: It is postulated that B-cell depletion by RTX may have removed an unknown regulatory effect of B-cells on T-cells leading to T-cell activation [14]. RTX may also lead to impairment of the response to infection, which may trigger plaque development [4, 9]. Another popular theory is that RTX can induce autoimmune phenomena, one of the manifestations of which could be immune-related skin lesions [4]. However, Thomas et al. reported the incidence rate of new onset psoriasis on 1927 RA patients who received RTX to be 1.04/1000 patient-years (95% CI 0.13 to 3.8), making it a rare complication [15]. In contrast to TNF-alpha inhibitor, a systematic review published in 2017 demonstrated 216 cases of new onset psoriasis induced by use for Crohn's disease (41% of cases), rheumatoid arthritis (37%), and ankylosing spondylitis (14%) [16].

In conclusion, new onset of psoriasis related to certain drug exposure is not uncommon. However, the incidence after the exposure to RTX is considered a rare side effect. A hypothesis explaining these interactions is still obscure, and a subject of debate is whether withdrawal of the offending drug alone is sufficient in terms of the recurrence and outcome.
